# Human Immunodeficiency Virus type 1 in seronegative infants born to HIV-1-infected mothers

**DOI:** 10.1186/1743-422X-3-52

**Published:** 2006-06-29

**Authors:** Vázquez Pérez JA, Basualdo Sigales MC, Reyes-Terán G, Gudiño Rosales JC, Soler Claudín C

**Affiliations:** 1 Unidad de Servicios Para Diagnóstico y Referencia en VIH, Instituto de Investigaciones Biomédicas UNAM/Secretaría de Salud del DF, México D.F; 2 Centro de Investigaciones en Enfermedades Infecciosas, Instituto Nacional de Enfermedades Respiratorias, México, D.F. Calzada de Tlalpan 4502 Mexico D.F; 3 Instituto de Diagnóstico y Referencia Epidemiológicos, Secretaría de Salud, México D.F. Carpio 470 Sto Tomás México D.F; 4 Benjamin Hill 24 Col Condesa, Miguel Hidalgo 06100 México D.F

## Abstract

**Background:**

Some individuals repeatedly exposed to Human Immunodeficiency Virus do not seroconvert and are resistant to HIV infection. Here, in a pediatric cohort of HIV seronegative infants born of HIV-infected mothers, we have studied eight non-breastfed children in whom viral DNA was detected in their PBMC. Our objective was to assess whether silent infection in these children can be explained by the presence of integrated viral DNA.

**Methods:**

The presence of viral DNA was corroborated by nested PCR with primers for *gag *and the *nef*/LTR regions of HIV-1. Integration of HIV DNA into the host genome was assessed by an Alu-LTR PCR. Amplicons were sequenced and phylogenetic analyzes were done.

**Results:**

HIV-1 DNA was detected in the earliest available PBMC sample from all eight infants, and two of them tested positive for HIV DNA at 2 years of age. Nested PCR resulted in the amplification of *gag*, nef/LTR and Alu-LTR fragments, which demostrated that HIV-1 DNA was integrated in the host cell genome. Each individual has a characteristic sequence pattern and is different from the LTR sequence of HXB2 prototype virus and other Mexican isolates.

**Conclusion:**

HIV-1 DNA was observed in PBMC from HIV exposed seronegative children in this pediatric cohort.

## Background

Several studies have shown that some individuals repeatedly exposed to Human Immunodeficiency Virus Type 1 or its antigens are resistant to HIV infection [1-6]. Despite multiple exposures to HIV, several of these resistant subjects have no detectable anti-HIV IgG antibodies in serum but instead present high anti-HIV CD4+ cell lymphoproliferative activity and strong CD8 cell mediated antiviral responses [1,2]. Other studies have described rare cases of HIV-1 exposed seronegative individuals (ES) in whom HIV DNA has been detected in peripheral blood cells by PCR. These exposed seronegative individuals include health care personnel with accidental percutaneous exposure to infected blood, sexual partners of known HIV-1-infected persons and infants born to HIV-1-infected mothers [3-6]. In case of the pediatric infections some of these children appear to have eliminated virus-infected cells [3], others continue to harbor cells with viral DNA for prolonged periods of time [4]. These antibody-negative, HIV-1 DNA-positive children have also been called "silent pediatric infections".

In this study, we report silent pediatric infection in 8 children born from HIV-1-positive mothers. Viral DNA could be amplified from their PBMC but we observed no evidence of viral replication or anti-HIV IgG antibodies in serum.

## Methods

To examine the potential presence of HIV DNA in seronegative children born to HIV-1-infected mothers of the Mexico City Reference and Diagnostic Unit HIV Pediatric Cohort, we selected 8 children on the basis of repeatedly negative virus culture and a positive HIV DNA PCR result in our laboratory. The children did not show any HIV/AIDS related symptoms and had never received antiretroviral treatment. Blood plasma HIV-1 RNA concentration (viral load) was negative in any children samples. Stored blood samples from each child were studied at different ages (Table 1). The study received approval of the Committee for Human Subject Research (Ministry of Health of México).

**Table 1:** Detection of HIV-1 LTR and GAG fragments in PBMC from seronegative infants born to HIV-1 infected mothers and controls.

Subject	No. Sample	Age (Months)	HIV antibodies	PCR		Integrated DNA
				LTR	GAG	Alu-LTR

P1	a	14	Negative	+	+	+
	b	15	Negative	+	+	+
	c	22	Negative	+	+	+
						
P2	a	3	Negative	+	+	+
	b	6	Negative	+	+	+
	c	11	Negative	-	-	-
	d	22	Negative	-	-	-
						
P3	a	18	Negative	+	+	+
	b	21	Negative	+	+	+
	c	29	Negative	-	-	-
						
P4	a	15	Negative	+	+	+
						
P5	a	16	Negative	+	+	+
	b	24	Negative	+	+	+
						
P6	a	24	Negative	+	+	+
	b	55	Negative	-	-	-
						
P7	a	20	Negative	+	+	+
	b	24	Negative	-	-	-
	c	29	Negative	-	-	-
						
P8	a	11	Negative	+	+	+
	b	14	Negative	-	-	-
						
NI*	NA	NA	Negative	-	-	-
IIIB°	NA	NA	NA	+	+	+
PINF°	NA	NA	Positive	+	+	+

*PBMC of noninfected children (NI) were used as a negative control.

°IIIBMolt cells and PBMC of HIV infected children (PINF) were used as a positive control in each experiment.

**Figure 1:**
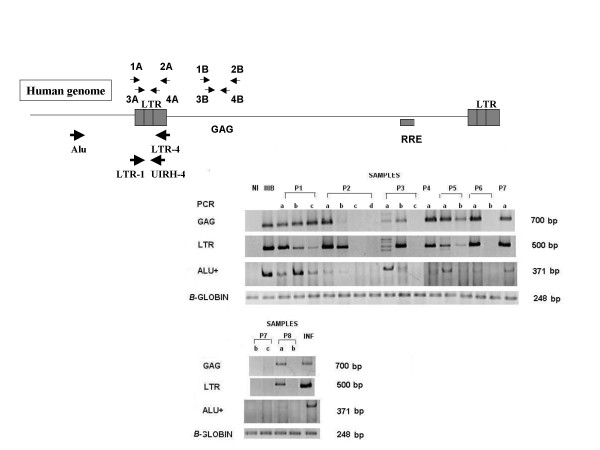
Integrated HIV DNA in PBMC of Seronegative Children. (A) Schematic representation of PCR amplification of the HIV proviral genome. Primers used for detection of LTR (1A to 4A) and GAG (1B to 4B) fragments are indicated by small thin arrows. PCR amplifications (Alu/LTR-4) of existing Alu-HIV LTR junctions were subjected to a second round of PCR with HIV-1 LTR-specific primers LTR-1-UIRH-4 (thick arrows). (B) PCR amplifications from PBMC DNA of samples children: P1 (a-c), P2 (a-d), P3 (a-c), P4 (a), P5 (a-b), P6 (a-b), P7 (a-c) and P8 (a,b). IIIBMolt cells and PBMC of HIV infected children (PINF) were used as a positive control. PBMC of noninfected children (NI) were used as a negative control.

To detect HIV-1 sequences in PBMC, two nested polymerase PCR amplifications were used (GAG and nef/LTR). The initial amplification of DNA was performed using GAG1-GAG2 (5'TCCACCTATCCCAGTAGGAG3' and 5'GGTCGTTGCCAAAGAGTGAT3') or LTR1-LTR2 primers [7]. An aliquot (5 μL) of first round PCR product was then used as a template in a second PCR reaction with GAG3-GAG4 (5'TAAAAGATGGATAATCCTGGG and 5'GCCAAAGAGTGATCTGAGGG3') or LTR3-LTR4 primers [7]. Controls for contamination (DNA of seronegative children) and for sensitivity (10 HIV copies) was added in each experiment in order to exclude all non-sensitive experiments. Different rooms were used for DNA extraction, PCR-buffer preparation, amplification and electrophoresis. Amplicons were never transferred to the area reserved for unamplified sequences. Thus, we cannot attribute positive PCR results to contamination. To detect integrated HIV-1 DNA, 2 μg of DNA were subjected to amplification by Alu-LTR PCR [8], using the Alu primer and HIV-1 LTR primer LTR4. After Alu-LTR PCR, a second round of PCR was performed with an aliquot equivalent to 1/10 of the PCR products using LTR specific primer pair LTR3-UIRH4 (Fig 1). To examine the LTR/nef region, PCR amplicons of the second round (LTR3-LTR4) were sequenced [7].

Phylogenetic relationships were determined using the MEGA2 versión 3.1 software package. A Phylogenetic tree was constructed by the neighbor-joining method and tree was bootstrapped with 100 replications. We used the Kimura two-parameter model to calculate sequence variation within the LTR sequences.

## Results

### Detection of HIV-1 DNA in exposed seronegative children

HIV-1 DNA was detected by the amplification of GAG and LTR fragments (Fig. 1). Based on these results, two groups of patients were observed: in the first group (children P1 and P5), persistent detection of both fragments (GAG and LTR) in sequential samples were detected; in the second group (children P2, P3, P4, P6, P7 and P8), in whom GAG and LTR fragments were detected in early samples, these viral genes were not detected in subsequent samples.

### Integration of the HIV-1 genome detected in exposed seronegative children

All the PBMC samples with a positive gag and LTR fragment amplification also resulted in amplification of the Alu-LTR binding sequence (Fig. 1). These results confirmed the presence of HIV DNA and indicated that the viral DNA detected in the children was integrated into the host genome. Additionally total mRNA was extracted from PBMC and HIV-1 mRNA transcripts were amplified by reverse-transcriptase PCR (data not shown). The complete or unprocessed mRNA was not detected in any children samples.

### *nef*/LTR sequences of eight children have a characteristic sequence pattern

**Figure 2:**
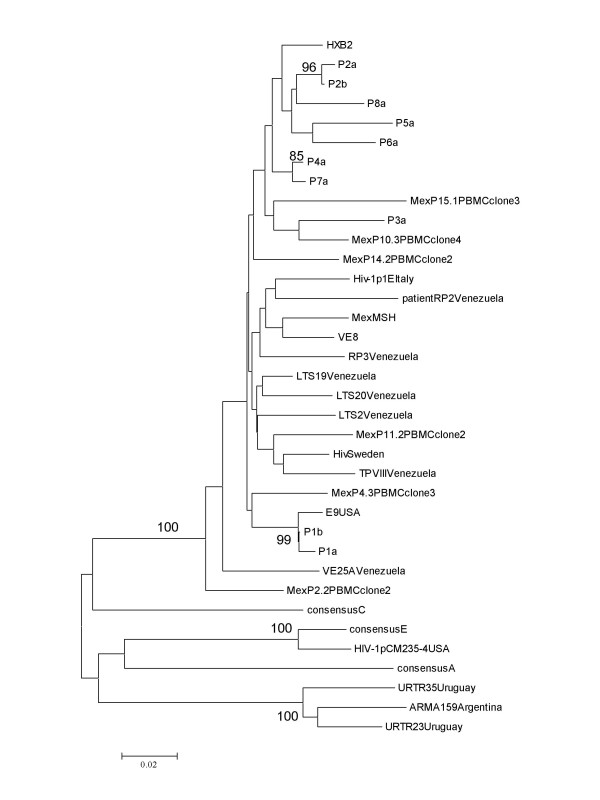
Phylogenetic relationship of LTR sequences from pediatric cohort of HIV seronegative infants. A neighbor-joining phylogenetic tree was generated from LTR sequences. Numbers at branch nodes indicate bootstrap proportions greater than 70 out of 100 bootstraps replicates. Kimura two-parameter method of estimating genetic distances was used. The patient identification is at the end of each corresponding branch. The LTR sequences of Mexican isolates (7), subtype B isolates and the consensus A, B (HXB2), C and E were included .

The *nef/*LTR regulatory region sequence in the proviral DNA of the children's cells was obtained and compared to that of HXB2 prototype virus and consensus sequences of clades of A, C, and E. All sequences were shown by phylogeny to be more similar to the consensus of clade B (Fig 2). Each individual has a characteristic sequence pattern and is different from the LTR sequence of HXB2 prototype virus and other Mexican isolates [7]. Sequential analysis of *nef*/LTR sequences of samples of P1 and P2 children were done and Phylogenetic relationship were estimated. Sequences of sequential samples of P1 and P2 were grouped closely in the same cluster, supporting the closely relationship between these samples (fig 2). A variation of 4% was observed in the region overlapping the *nef *gene, between -295 and -121 bp. In contrast, the non-translated region, between -120 and +80 bp, presented a variation lower than 2%. These results are similar to data reported in other studies with AIDS patients [9].

## Discussion

Cases of children who appear to have eliminated HIV infection are rare as most of them reported are due to contamination in the PCR processes. Moreover, these previous studies analyzed only *env *sequences of viruses isolated from children with apparent silent infections and their seropositive mothers, and no phylogenetic relation was found among the isolates [10]. In our study, the presence of the viral genome was confirmed by amplification of two different sequences in the conserved Gag/LTR regions of the virus. Additionally, PCR contamination in our study is improbable since DNA extraction, first and second round amplification and separation of PCR products by electrophoresis were performed in different areas. Negative control of DNA of Non-Infected PBMC was included in every reaction and no amplification was shown (Fig 1).

It is also unlikely that cells from the mother were being a source of contamination. It has been shown that the passage of infectious agents or cells to the fetus is rather limited [11]. Even though there is some evidence that bidirectional traffic of cells, including leukocytes, may occur in human pregnancy [12], the frequency of such traffic determined was not consistent in previous studies [13]. Furthermore, in a mouse model the maternal cells were undetectable after 9 days postpartum [13]. In our case the earliest sample was taken at 3 month of age, and thus the presence of mother cells at this time in the children is improbable. Unfortunately It was not possible to analyze the sequence of the virus of the children's mothers in order to confirm relatedness. Nevertheless sequences of the *nef/*LTR region of the viruses analyzed showed HIV genetic diversity in our cohort among the children and with other Mexican virus isolates [7]. Additionally we demonstrate phylogenetic relationship between the sequential samples of two children P1 and P2. This agrees with the amplification of proviral DNA that comes from the subject, and makes less likely the possibility of contamination.

## Conclusion

Our results indicate that the seronegative children studied here were exposed to HIV and had cells with proviral HIV. The particularly long period of time of detection of the viral genome suggests that proviral HIV can be present in cells with a very long half-life, probably in resting CD4+ T cells that keep HIV suppressed. Moreover we find no evidence of active or productive infection consistent of HIV viral load negative results and no detection of unspliced HIV RNA (data not shown) in any children sample. This indicates the possibility of dead end infection in patients that loose proviral DNA. These results suggests an extends revision in molecular diagnostic of HIV children infection specific in PCR of HIV DNA from peripheral blood mononuclear cell (PBMC), and detection of virus by PBMC co-culture in peripheral blood from the infant.

## Competing interests

The author(s) declare that they have no competing interests.

## Authors' contributions

Gudiño Rosales JC helped to carried out the molecular genetic studies. Basualdo Sigales MC carried out the virus culture of the Mexico City Reference and Diagnostic Unit HIV Pediatric Cohort. Reyes Teran G. participated in the design of the study. Soler C. conceived of the study, and participated in its design and coordination and helped to draft the manuscript. Vazquez Perez JA carried out the molecular genetic studies, participated in design of the study and coordination and drafts the manuscript. All authors read and approved the final manuscript.
